# Reduced Retinal Thickness Predicts Age-Related Changes in Cognitive Function

**DOI:** 10.3389/fnagi.2020.00081

**Published:** 2020-03-24

**Authors:** Najiba Mammadova, Tricia K. Neppl, Natalie L. Denburg, M. Heather West Greenlee

**Affiliations:** ^1^Virus and Prion Research Unit, National Animal Disease Center, USDA, Agricultural Research Service, Ames, IA, United States; ^2^Department of Human Development and Family Studies, Iowa State University, Ames, IA, United States; ^3^Department of Neurology, University of Iowa, Iowa City, IA, United States; ^4^Department of Biomedical Sciences, College of Veterinary Medicine, Iowa State University, Ames, IA, United States

**Keywords:** retina, retinal nerve fiber layer, optical coherence tomograhy, cognitive function, executive function

## Abstract

Currently, there is a lack of biomarkers to identify individuals in the early stages of Alzheimer’s disease (AD). A preponderance of evidence suggests that neurodegenerative processes that affect the brain, may also affect the retina. Using optical coherence tomography (OCT), a non-invasive approach, many have shown thinning of the retina in AD and the developmental precursor to AD, *mild cognitive impairment* (*MCI*). However, the relationship between retinal thickness and cognitive function is not entirely clear. This is likely due to the disparity in diagnostic criteria used to determine MCI that does not fully probe the cognitive domains that are particularly vulnerable to aging. This study used a comprehensive neuropsychological assessment involving multiple domains of cognition to determine if retinal thickness correlates with cognitive performance in a normal aged population. In this study, 20 healthy individuals between 60 and 90 years of age were administered neuropsychological assessments probing various domains of cognitive function, and OCT to measure peripapillary retinal nerve fiber layer (RNFL) thickness. We found that RNFL thickness is correlated with neuropsychological performance in multiple cognitive domains (e.g., working memory, psychomotor speed, and executive function). Our work demonstrates a positive correlation between RNFL thickness and several, but not all, domains of cognitive function in a normative aging population. By determining which cognitive domains retinal thickness can predict, this work can help identify individuals at risk or in preclinical stages of AD and other neurodegenerative diseases.

## Introduction

Alzheimer’s disease (AD) is clinically characterized by a progressive decline in episodic memory and cognition, as well as deficits in executive functioning and learning (Gao et al., 2015). Currently, clinical diagnosis and staging of AD requires invasive or expensive techniques that have been contraindicated due to a high risk/benefit ratio, whereas definitive diagnosis of AD can only be made at brain autopsy (Beach et al., 2012). Thus, along with continued efforts to understand disease mechanisms, and provide therapies at the earliest stage of the disease process, there is a need to develop sensitive and less invasive diagnostic approaches to identify individuals at risk for AD. Thus, there is clinical and epidemiological emphasis on the developmental precursor to AD, entitled mild cognitive impairment (MCI) due to AD, as a way to identify early changes in subjects at risk for AD (Knoll et al., 2016). MCI, specifically the amnestic subtype, consists of isolated cognitive deficits on neuropsychological assessment of anterograde memory, but no impairment of daily activities, thus representing the pre-clinical phase of AD (Knoll et al., 2016). Prominent visual deficits including contrast sensitivity and poor visual memory performance have been reported in AD patients years before clinical diagnosis, with increasing evidence of pathology at several levels of the visual pathway (Gao et al., 2015). Optical coherence tomography (OCT) has emerged as a non-invasive and readily available approach to observe changes in retinal structure, providing high-resolution cross-sectional images of the macula, and retinal nerve fiber layer (RNFL) (Cheung et al., 2017; Doustar et al., 2017). The RNFL is formed by retinal ganglion cells axons and represents the innermost layer of the retina. To date, many groups have used OCT to demonstrate thinning of the RNFL in patients with AD (Lu et al., 2010; Kesler et al., 2011; Moschos et al., 2012; Ascaso et al., 2014; Liu et al., 2015; Thomson et al., 2015; Den Haan et al., 2017). While meta-analyses demonstrate consistent retinal thinning in patients with AD, the relationship between retinal change and MCI is not as clear. This may perhaps be due to methodological differences in the diagnostic criteria used to determine MCI that are currently restricted to brief screening tests such as the Mini Mental Status Examination (MMSE), and therefore do not fully probe cognitive skills vulnerable to aging (i.e., episodic memory, language, and others) (Folstein et al., 1975; Buckner, 2004; Petersen et al., 2014). Few studies have used comprehensive neuropsychological assessments to describe an association between retinal structure and cognitive function in population-based samples of healthy subjects. Van Koolwijk et al. (2009) assessed several domains of cognitive functioning in a large population of healthy individuals, and reported a significant association between a thicker RNFL and better cognitive performance. More recently, Ko et al. (2018) reported a significant correlation between RNFL thickness, derived using OCT, and cognitive function in a normative population (range, 40–69 years of age). This work used a range of tests to assess *general* cognitive functions including reaction time or processing speed, and numeric and verbal reasoning (Ko et al., 2018). However, Ko et al. (2018) did not investigate a correlation between specific cognitive domains and RNFL thickness. Identification of the specific cognitive domain(s) correlated with retinal thickness will help to determine the utility of retinal measurements in predicting cognitive decline. In this study, we use a comprehensive neuropsychological assessment involving multiple domains of cognition (working memory, psychomotor speed, executive function, visuospatial abilities, intellect, language, and memory) and OCT to determine if retinal thickness correlates with cognitive performance in specific domains, in a normal aging sample. By determining how retinal thickness correlates with cognitive function in a healthy aging population, we address the limitations of previous studies in regard to methodology and reliability of biomarkers to identify individuals at risk for MCI and AD.

## Materials and Methods

### Participants

As part of a larger, ongoing project, healthy older adults (*N* = 20; Median*_*age*_* = 73.4 ± 5.0; range = 60–90 years; 50% female) were recruited from churches, synagogues, and senior organizations and clubs. All participants lived independently in the community. A semi-structured interview, which assessed neurological status, current medications, alcohol/drug consumption, and mood (Tranel et al., 1997), was conducted individually with each participant to ensure all were healthy. Participants in the current study were independently living, community-dwelling, and cognitively intact, with no history of neurological or psychiatric disease as determined secondary to extensive clinical interview (Tranel et al., 1997). More specifically, prior to participation in this study, all participants were administered a health interview to exclude individuals with any outstanding medical or psychiatric conditions (e.g., stroke, head injury with extended loss of consciousness, Type I diabetes, neurosurgery, seizure disorder, demyelinating disorder, substance abuse, uncontrolled medical condition, vision/hearing loss, psychiatric illness necessitating inpatient treatment, and self-reported depression and/or anxiety exceeding mild severity). Additionally, neuropsychological data was used to screen out individuals with cognitive impairments, secondary to falling outside the normal range (1.5 standard deviations) for their age, level of education, and premorbid intellect (Lezak et al., 2012). Participants were compensated for their participation in the study at an hourly rate.

### Neuropsychological Assessment and Retinal Measurements

Mental status screening was conducted with the Mini-Mental State Examination (Folstein et al., 1975), a 30-point, verbally administered questionnaire sensitive to impairment in orientation, word recall, attention, calculation, language, and visuospatial ability. *Current intellectual ability* was measured using the Wechsler Abbreviated Scale of Intelligence-Second Edition (WASI) (Wechsler, 1997, 2008; Wechsler and Hsiao-pin, 2011). Full scale IQ, which consists of four subtests (two verbal and two non-verbal): Vocabulary, Similarities, Block Design, and Matrix Reasoning. *Language* was assessed with The Boston Naming Test (Goodglass et al., 2000) and the Delis-Kaplan Executive Function System Verbal Fluency (Delis et al., 2001). The Boston Naming Test was used to systemically examine the fast and accurate oral production of words necessary for the naming of simple line drawings. The D-KEFS Verbal Fluency consists of three conditions. We utilized Letter Fluency (Condition 1), which required the participant to name as many words as they could beginning with a particular letter (first F, then A, and then S), during a one-minute interval. We also utilized Category Fluency (Condition 2), which required the participant to name as many words as they could that fulfilled a category rule (first animals, then boy names) during a one-minute interval. *Anterograde memory* was assessed with the Rey-Osterrieth Complex Figure (Lezak et al., 1995), which was used to evaluate non-verbal memory during the 30-min delay condition. The Rey Auditory Verbal Learning Test (Rey, 1941) was administered as a measure of verbal learning and memory, providing a measure of 30-min delayed memory. *Attention and concentration*, *but more specifically working memory* were assessed with the Digit Span Forward and Backward subtests, respectively, of the Wechsler Adult Intelligence Scale-Fourth Edition (WAIS) (Wechsler, 1997). *Visuospatial skills* were assessed with the copy condition of the Rey-Osterrieth Complex Figure test (Lezak et al., 2004) and the Judgment of Line Orientation Test (Benton et al., 1994), a measure of visuospatial judgments. Psychomotor speed was assessed with the Coding subtest of the WAIS-IV (Wechsler, 1997) a measure of clerical speed and accuracy, and with the Trail Making Test 2 of the Delis-Kaplan Executive Functioning System (Delis et al., 2001), identical to the classic Trail Making Test Form A. We utilized Number Sequencing (Condition 2, akin to the well-known Trails A), a measure of simple motor speed, in which the participant draws a line to connect numbers 1–16 in numerical order, as quickly and accurately as possible. *Executive functioning* was assessed with the Trail Making Test 4 of the Delis-Kaplan Executive Functioning System (Delis et al., 2001). We utilized time (in seconds) taken to complete TMT 4 of the D-KEFS, which is identical to the classic Trail Making Test Form B, a measure of speeded set-shifting. The TMT consists of five conditions. We utilized Number-Letter Switching (Condition 4, akin to the well-known Trails B), a measure of executive functioning, in which the participant switches back and forth between connecting numbers and letters (i.e., 1 to A, A to 2, and 2 to B, etc.), as quickly and accurately as possible. Outcome for the WASI Coding and DKEFS Trails 2 and 4 assessments is the time to complete the tasks, therefore lower values indicate better performance. The D-KEFS Tower Test provides the participant with five discs of different sizes, and three “towers” (vertical rods). The participant is given instructions to move the discs from the start position to the finish position in as few moves as possible and following certain rules (e.g., cannot place a larger disc on a smaller disc).

In brief, high-resolution spectral-domain OCT imaging of undilated eyes was performed by a trained technician and reviewed by a neuro-ophthalmologist using the Zeiss Cirrus 5000 HD-OCT (Version 7.5.0.56), on the same day as other physical measurements. RNFL thicknesses were obtained using the Optic Disc Cube 200 × 200 program, with 200A scans from 200 linear B-scans evenly distributed in a 6 mm^2^ distance over the center of the optic nerve head. The minimum acceptable signal strength score was 6. Based on specific RNFL layer boundaries, the RNFL thickness at each point along the calculation circle was calculated. Using this, Cirrus OCT provided the 12-clock-hour thicknesses, four quadrant thicknesses, a global 360° average thickness, and TSNIT thickness profiles (described in Jeoung and Park, 2010). One-tailed partial correlations were conducted to examine the associations between RNFL thickness and the neuropsychological variables, while controlling for age. No *post hoc* test was conducted to correct for multiple comparisons. A *p*-value less than 0.05 was considered statistically significant. All statistical data analyses were performed using SPSS version 25.0 (IBM Corp, 2017).

## Results

To assess the correlation between RNFL thickness and cognitive function, 20 healthy older adults were administered a neuropsychological battery (see section “Materials and Methods”) and OCT yielding a measurement for each of four quadrants (superior, temporal, inferior, and nasal) for both eyes and a “total” average of all quadrants. The mean age of this sample was 73.4 ± 5.0 years, mean education was 16.1 ± 2.8 years, and mean Mini Mental Status Exam (MMSE) score was 29.2 ± 1.2. Table 1 represents regression coefficients/*p*-values from all cognitive domains tested. Our results showed significant positive correlations between RNFL thickness of *the right and left eyes* and neuropsychological performance in multiple cognitive domains. WAIS Digit Span B. refers to the Digit Span Backward subtest of the WAIS-IV used to assess working memory. There was a significant correlation between WAIS Digit Span Backward and the RNFL temporal quadrants of both eyes (Table 1). D-KEFS Trails 2 was used to assess psychomotor speed and visuomotor tracking, while Trails 4 is sensitive to executive functioning and visuomotor tracking (Reitan and Wolfson, 1985). There was a significant correlation between psychomotor speed and executive functioning, and the RNFL inferior quadrants of both eyes, as well as the average regression coefficients of all four quadrants in both eyes (Table 1).

**TABLE 1:** Regression coefficients (R) and *P* values in parentheses correlating peripapillary RNFL thickness to cognitive function in a healthy aging population, *N* = 20.

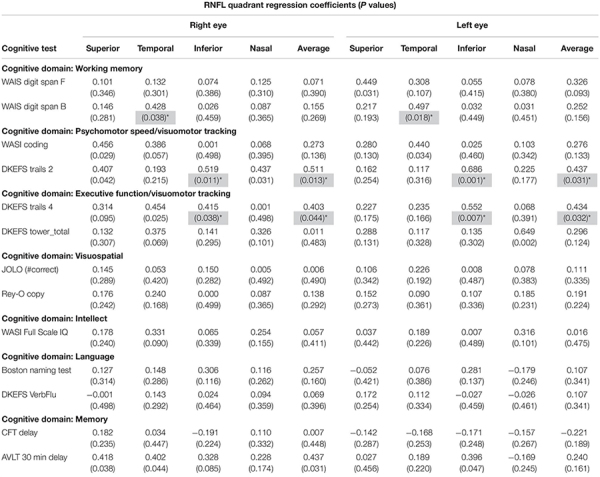

*Correlation is statistically significant (<0.05). Shaded values represent *p*-values that are significant in similar quadrants of the right and left eyes.

## Discussion

Our results demonstrate significant correlations between thickness of the RNFL and specific cognitive functions in a healthy aging population, which has not been previously reported. In this study we take into consideration the limitation of the small sample size, as well as not statistically correcting for multiple comparisons. However, while we report regression coefficients/*p*-values for all comparisons, we only highlight planned comparisons (i.e., regression coefficients/*p*-values that were significant in similar quadrants in the right and left eyes) as a way of ameliorating the issue of multiple comparisons (Rothman, 1990; Saville, 1990). We show a positive correlation between RNFL thickness of the right and left eyes and neuropsychological performance in the WAIS Digit Span Backward subtest and the D-KEFS Trails 2 and 4. The WAIS Digit Span Backward subtest was used to assess working memory and selective attention, while D-KEFS Trails 2 and 4 were used to assess psychomotor speed, cognitive flexibility and executive function, respectively. Cognitive performance in these three tests is sensitive to prefrontal cortex function, therefore these findings may suggest an association between thinning of the RNFL and cognitive decline with involvement of the prefrontal cortex. We also note that there was not a significant positive correlation between RNFL thickness of the right and left eyes and neuropsychological performance in the D-KEFS Tower Test, which probes spatial planning and rule learning that are also sensitive to prefrontal cortex function. Additionally, we found no significant correlations between RNFL thickness and other domains of cognitive function, primarily visuospatial skills, general, and verbal intelligence as well as episodic memory commonly associated with functioning of the temporal parietal junction. Having said that, we take into consideration the limitation of the small sample size, and reiterate that the more fine-grained nature of the relationship between RNFL thickness and cognitive function needs to be further investigated.

Retinal thinning, specifically thinning of the RNFL, in MCI and AD patients has been demonstrated by several studies, however, there are discrepancies regarding whether there is a correlation between retinal changes and the severity of dementia (Lu et al., 2010; Kesler et al., 2011; Ascaso et al., 2014; Gao et al., 2015; Liu et al., 2015; Thomson et al., 2015; Den Haan et al., 2017). There is also no consensus regarding MCI or AD associated RNFL thinning of specific retinal quadrants, however, majority of reports show selective thinning of the RNFL in the superior and/or inferior quadrant (Berisha et al., 2007; Chi et al., 2010; Kirbas et al., 2013). While there is a lot of interest in establishing the relationship between dementia and retinal thickness as a tool for early diagnosis, there is a lack of consistent or predictive data in current literature. A hindrance to the development of therapies to prevent dementias is the lack of biomarkers that can predict individuals at risk. Moreover, cognitive function tests in many studies are often restricted to the MMSE, which does not probe the full extent of cognitive domains, that are vulnerable to aging, that may also be affected in MCI or AD. This work assesses the relationship between RNFL thickness of specific quadrants and multiple cognitive domains in a normal aging population, using a comprehensive neuropsychological assessment (Table 1). Van Koolwijk et al. (2009) assessed the relationship between several domains of cognitive functioning (i.e., general cognitive ability using a reading test, short-term memory and delayed recall, executive functioning, and visuospatial abilities) and retinal structure (evaluated using scanning laser polarimetry) in a large population of healthy individuals (range, 18–85 years of age) (Van Koolwijk et al., 2009), and reported a significant association between a thicker RNFL and better cognitive performance, however, this correlation diminished in individuals beyond 40 years of age. More recently, another study used OCT to identify a positive correlation between RNFL thickness and current and future cognitive decline in a large community cohort of healthy people, however, an association of specific domains of cognition was not identified (Ko et al., 2018). Overall, this collection of studies suggests the potential for OCT to predict future cognitive impairment, however, further studies with larger sample sizes are necessary to understand the underlying etiology, to support the use of evolving retinal imaging techniques as a useful tool in predicting individuals at risk for MCI and AD.

## Data Availability Statement

The datasets generated for this study are available on request to the corresponding author.

## Ethics Statement

The studies involving human participants were reviewed and approved by the University of Iowa Institutional Research Board (IRB). The patients/participants provided their written informed consent to participate in this study.

## Author Contributions

NM analyzed the data, prepared the figures, and wrote the manuscript. TN, ND, and MW analyzed the data, guided the study, contributed resources, and edited the manuscript.

## Conflict of Interest

The authors declare that the research was conducted in the absence of any commercial or financial relationships that could be construed as a potential conflict of interest.
